# The Prevalence of Vitiligo: A Meta-Analysis

**DOI:** 10.1371/journal.pone.0163806

**Published:** 2016-09-27

**Authors:** Yuhui Zhang, Yunfei Cai, Meihui Shi, Shibin Jiang, Shaoshan Cui, Yan Wu, Xing-Hua Gao, Hong-Duo Chen

**Affiliations:** 1 Department of Dermatology, No. 1 Hospital of China Medical University, Shenyang, 110001, China; 2 Department of Dermatology, No. 1 Hospital of Dalian Medical University, DaLian, 116011, China; Kinki Daigaku, JAPAN

## Abstract

**Objective:**

To conduct a meta-analysis assessing the prevalence of vitiligo.

**Methods:**

Literatures that reported prevalence rates of vitiligo were identified using EMBASE, PubMed, the Cochrane Library, China National Knowledge Infrastructure (CNKI), Wanfang database and Weipu database for the period from inception to May 2016. We performed stratified analyses on possible sources of bias, including areas difference, years of publication, gender and age. Publication bias was assessed with Egger’s test method.

**Results:**

A total of 103 studies were eligible for inclusion. The pooled prevalence of vitiligo from 82 population- or community-based studies was 0.2% (95%CI: 0.1%–0.2%) and from 22 hospital-based studies was 1.8% (95%CI: 1.4%–2.1%). A relatively high prevalence of vitiligo was found in Africa area and in female patients. For population- or community-based studies, the prevalence has maintained at a low level in recent 20 years and it has increased with age gradually. For hospital-based studies, the prevalence has showed a decreased trend from 60s till now or from young to old. No significant publication bias existed in hospital-based studies (t = 0.47, P = 0.643), while a significant publication bias existed in population- or community-based studies (t = 2.31, *P* = 0.026).

**Conclusion:**

A relatively high prevalence of vitiligo was found in Africa area and in female patients. The prevalence has maintained at a low level in recent years. It showed an inverse trend with age increment in population- or community-based studies and hospital-based studies.

## Introduction

Vitiligo refers to an acquired, idiopathic, and common de-pigmentation disorder of the skin [1]. The clinically characteristic symptoms of the vitiligo are pale or milk-white macules or patches due to the selective destruction of melanocytes. They occur on the skin in different parts of the body and sometimes also on the mucous membranes. The exact pathogenesis of vitiligo is still to be elucidated. Multiple mechanisms, including metabolic abnormalities, oxidative stress, generation of inflammatory mediators, cell detachment and autoimmune responses, might contribute to the pathogenesis. In particular, the autoimmune mechanism is now clearly established. Vitiligo may appear at any age and affect both sexes. It tends to occur or recur in spring and/or summer [2, 3].

Some previous reports on vitiligo epidemiology were based on population surveys, while others were performed in patients of dermatology clinics. However, the prevalence of vitiligo varies in different geographic regions and different sample size, and the data have limitations and localizations. Besides, the disorder afflicts various ethnic populations with varying prevalence estimates ranging from 0.1% to 2.0% based on the general populations in previous studies [4, 5]. But recently, some papers suggested that previous epidemiological data were exaggerated. To date, no meta-analysis on the prevalence of vitiligo has been conducted. Accordingly, it seems that an international and pooled estimate based on the various populations is necessary.

The main objective of this meta-analysis is to summarize all available data to give a description of a worldwide picture on the prevalence of vitiligo. The information was collected from both population- or community studies and hospital-based studies. Various epidemiological characteristics of vitiligo were studied in order to understand this disease more clearly.

## Materials and Methods

### Search Strategy

We conducted a systematic search of scientific databases, including EMBASE, PubMed, the Cochrane Library, China National Knowledge Infrastructure (CNKI), Wanfang database and Weipu database to find relevant papers published from inception to May 2016. The search medical subject heading (MeSH) terms and keywords were “vitiligo” OR “leucoderma” AND “prevalence” OR “epidemiology”. In addition, a manual search was supplemented by verifying a secondary review of the reference lists of key publications to confirm additional relevant citations.

### Inclusion and Exclusion Criteria

The criteria of included studies were as follows: (1) had sufficient information to estimate the pooled prevalence of vitiligo; (2) population-based, community-based or hospital-based; (3) published in either English or Chinese language.

The exclusion criteria of studies were: (1) irrelevant to vitiligo; (2) irrelevant to our topic; (3) review; (4) duplicate data.

### Data Extraction

The whole potentially relevant information from the included studies was independently reviewed by two investigators (Yuhui Zhang, Meihui Shi) using a standardized form which was designed in advance. When there was a disagreement about whether selecting articles should be resolved for analysis or not, a third investigator (Yunfei Cai) made the final decision. The following information was extracted from each suitable study: first author’s name, years of publication, country, survey age, gender of the participants, survey year, total sample size, numbers of vitiligo and prevalence rate.

### Data Analysis

All statistical analyses were made using Stata software (version 12.0; Stata Corporation, College Station, Texas, USA) and the meta package was used to produce the pooled estimates, forest plots and publication bias assessment. Initially, the pooled prevalence estimates of vitiligo and 95% confidence intervals (CIs) were calculated assuming a fixed-effect model when significant heterogeneity was absent (*P*>0.1, I^2^<50%). If significant heterogeneity was present (*P*<0.1, I^2^>50%), a random-effect model was selected. To determine possible causes of heterogeneity, subgroup analyses were conducted by areas, years of publication, gender, and age. The areas covered Asia (India, China, Saudi Arabia, Sri Lanka, Turkey, Nepal, Iran, Korea, Kuwait, Thailand, Japan, Jordan), Africa (Tanzania, Egypt, Mali, Mozambique, Nigeria, Congo), America (USA, Brazil, Mexico, West Indies), Europe (Denmark, Sweden, Italy, Germany, Romania, France), Oceania (Australia) and Atlantic (Faroe Islands). For publication years, studies were grouped into eight periods, including 1 period before 80s and 7 periods after 80s with an interval of 5 years. For subgroup analysis according to age, it was grouped into four sections with an interval of 20 years. Publication bias was assessed by visually inspecting funnel plots and applying Egger’s tests to evaluate sources of variability. For all tests, *P* value < 0.1 was considered to be statistically significant.

## Results

### Literature search

**Fig 1:**
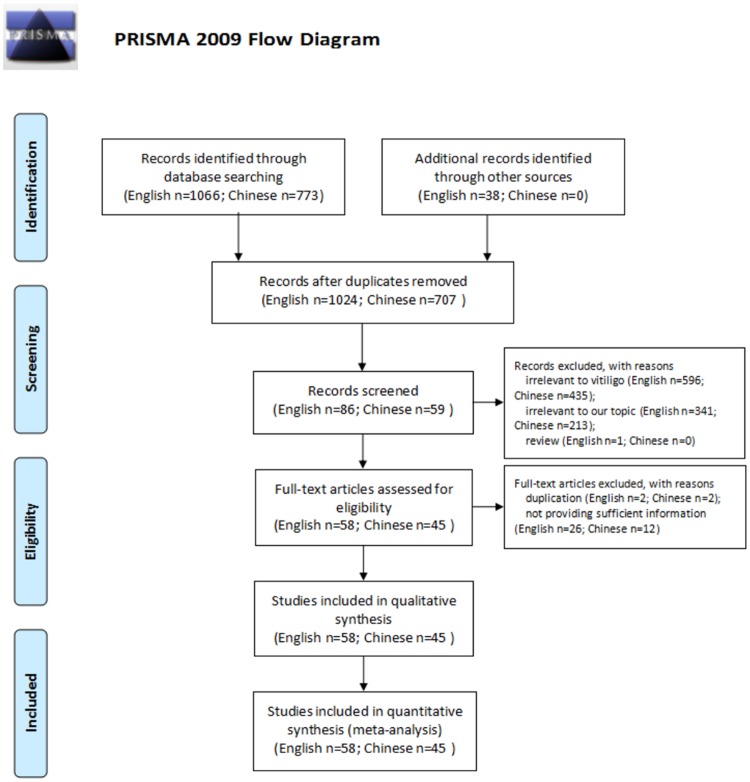
Flow diagram of the study selection process. Flow diagram of the study selection process.

A total of 1731 titles and/or abstracts of relevant studies were retrieved, and 1586 papers were removed due to irrelevance or review. The full-texts of remaining 145 papers were further reviewed, and 42 papers were excluded because of duplication and not providing sufficient information. Finally, 103 studies met the inclusion criteria and were included in this meta-analysis [6–108]. The flow chart of study selection process was shown in Fig 1.

### Study characteristics

Of the 103 studies, 82 were population- or community-based studies and 22 were hospital-based studies. The countries were Faroe Islands, India, Denmark, USA, Australia, Sweden, Brazil, China, Italy, Germany, Tanzania, Saudi Arabia, Romania, Sri Lanka, Egypt, France, Turkey, Mali, Mozambique, Nepal, Iran, Korea, Mexico, Kuwait, West Indies, Thailand, Nigeria, Japan, Congo, Jordan and the areas covered Asia, Africa, America, Europe, Oceania and Atlantic. The years of publication ranged from 1964 to 2015. The sample size of included studies ranged from 102 to 50593516. The prevalence of vitiligo ranged from 0.004% to 9.98%. The characteristics of included studies were summarized in Table 1.

**Table 1:** Characteristics of studies on the prevalence of vitiligo.

First Author	Publication Year	Country	Survey Age (years)	Survey Year	Sample (N)	Vitiligo (n)	Prevalence
Population or community-based studies							
Lomholt G [6]	1964	Faroe Islands	all	-	10984	7	0.06%
Mehta NR [7]	1973	India	all	1971~1972	9065	138	1.52%
Howitz J [8]	1977	Denmark	all	1971~1972	47033	179	0.38%
Johnson MT [9]	1978	USA	1~74	1971~1974	20749	102	0.49%
Quirk CJ [10]	1979	Australia	adults	-	1037	12	1.16%
Larsson PA [11]	1980	Sweden	12~17	-	8298	33	0.40%
Weismann K [12]	1980	Denmark	55~106	-	584	7	1.20%
Bechelli LM [13]	1981	Brazil	6~16	1974~1975	9955	4	0.04%
Zhou YH [14]	1985	China	all	1985	13390	1	0.01%
Das SK [15]	1985	India	≥3	1978~1982	15685	72	0.46%
Montagnani A [16]	1985	Italy	1month~12years	1979~1982	1273	12	0.94%
Nanda A [17]	1989	India	≤6 weeks	1986	310	1	0.32%
Schallreuter KU [18]	1991	Germany	14~86	1989	350	2	0.57%
Xue SQ [19]	1994	China	42~60	1992	5683	72	1.27%
Cellini A [20]	1994	Italy	23~79	1990~1992	526	2	0.38%
Wang WX [21]	1994	China	all	1984~1985	316379	294	0.09%
Gibbs S [22]	1996	Tanzania	all	-	1114	3	0.27%
Guan JC [23]	1997	China	15~20	1997	2206	1	0.05%
Bhatia V [24]	1997	India	0~14	1988~1989	666	4	0.60%
Kubeyinje EP [25]	1997	Saudi Arabia	18~45	1991~1995	1520	5	0.33%
Ren XL [26]	1998	China	-	-	155000	15	0.01%
Sun TQ [27]	1999	China	1~79	1996	78021	93	0.12%
Liao WQ [28]	1999	China	-	1997	3560	4	0.11%
Popescu R [29]	1999	Romania	6~12	1995	1114	3	0.27%
Perera A [30]	2000	Sri Lanka	all	1997	1806	22	1.22%
Ling WJ [31]	2001	China	-	1999~2000	102	1	0.98%
Xie PL [32]	2001	China	0~7	-	23052	1	0.004%
Che DF [33]	2001	China	-	1998~1999	3160	2	0.06%
Sun ZX [34]	2001	China	0~7	1999~2000	10804	2	0.02%
Zhang JQ [35]	2002	China	21~51	1999	641	3	0.47%
Zhang BX [36]	2002	China	17~31	1998~2000	3761	7	0.19%
Chen XQ [37]	2002	China	-	2001	11389	5	0.04%
Prahalad S [38]	2002	USA	-	-	496	2	0.40%
Yang XQ [39]	2002	China	16~24	2001~2002	2188	20	0.91%
El-Serag HB [40]	2002	USA	59.8113.41	1992~1999	136816	130	0.10%
Dogra S [41]	2003	India	6~14	2001	12586	272	2.16%
Abdel-Hafez K [42]	2003	Egypt	all	1994~1996	8008	98	1.22%
Xu YY [43]	2003	China	all	-	156461	279	0.18%
Li PH [44]	2003	China	all	2002	13953	3	0.02%
Wolkenstein P [45]	2003	France	all	2002	18137	51	0.28%
Zeng YH [46]	2004	China	6~12	2002	17542	6	0.03%
Feng D [47]	2004	China	17~58	2000	853	4	0.47%
Xu HZ [48]	2004	China	20~97	-	2195	1	0.05%
Zhao Y [49]	2004	China	18~24	2002	2116	1	0.05%
Naldi L [50]	2004	Italy	≥45	2003	3660	26	0.71%
Tuncel AA [51]	2005	Turkey	14~25	-	682	2	0.29%
Lin T [52]	2005	China	18~46	2004	385	1	0.26%
Faye O [53]	2005	Mali	<15	2001	1729	4	0.23%
Wang TL [54]	2006	China	17.4~23.8	2004	34166	35	0.10%
Song WF [55]	2006	China	-	2005	3920	2	0.05%
Ai JZ [56]	2006	China	7~16	-	21794	3	0.01%
Al-Saeed WY [57]	2006	Saudi Arabia	6~17	2003	2239	8	0.36%
Lu T [58]	2007	China	all	2002~2003	42833	40	0.09%
Zhao G [59]	2007	China	18~44	2006	324	3	0.93%
Xu CY [60]	2007	China	-	2007	4725	3	0.06%
Chhaganlal K [61]	2007	Mozambique	0~82	3-month period	780	1	0.13%
El-Essawi D [62]	2007	USA	20~80	-	194	3	1.55%
Chen GY [63]	2008	China	6~11	2005	3273	3	0.09%
Birlea SA [64]	2008	Romania	all	2001~2006	2021	3	0.15%
Walker SL [65]	2008	Nepal	12 days~80 years	-	878	8	0.91%
Zhao G [66]	2009	China	22~57	2007~2008	255	1	0.39%
Zhu LB [67]	2009	China	all	2007~2008	6593	2	0.03%
Komba EV [68]	2010	Tanzania	6~19	-	420	3	0.71%
Liu XH [69]	2010	China	-	2007	1670	1	0.06%
Li YF [70]	2010	China	18~53	2008	1078	3	0.28%
Ingordo V [71]	2011	Italy	18	2001~2004	34740	60	0.17%
Pei GD [72]	2011	China	18~94	-	2341	22	0.94%
Wang RL [73]	2012	China	12~20	2008~2009	7747	37	0.48%
Yamamah GA [74]	2012	Egypt	≤18	2008~2009	2194	4	0.18%
Liu Q [75]	2013	China	12~80	-	2719	21	0.77%
Wang XY [76]	2013	China	all	-	17345	122	0.70%
Yang YS [77]	2013	China	17~21	-	1525	2	0.13%
Pang XW [78]	2013	China	18~39	2011	473	3	0.63%
Chen JZ [79]	2013	China	17~21	-	2957	2	0.07%
Zhu XW [80]	2014	China	18~93	2012	3993	2	0.05%
Shao ZQ [81]	2014	China	-	2011	986	1	0.10%
El-Khateeb EA [82]	2014	Egypt	6~12	2011~2012	6162	4	0.06%
Reddy J [83]	2014	India	all	-	22037	160	0.73%
Afkhami-Ardekani M [84]	2014	Iran	10~98	2011	1100	20	1.82%
Lee H [85]	2015	Korea	all	2009~2011	50593516	63467	0.13%
Chen YT [86]	2015	China	all	1997~2011	23254688	14883	0.06%
Liu TH [87]	2015	China	17~43	2014	1347	2	0.15%
Hospital-based studies							
Ruiz-Maldonado R [88]	1977	Mexico	0~18	1971~1975	10000	260	2.60%
Anand IS [89]	1998	India	0~12	1994	400	8	2.00%
Nanda A [90]	1999	Kuwait	0~12	1992~1996	10000	149	1.49%
Boisseau-Garsaud AM [91]	2000	West Indies	1~96	1995~1996	2077	7	0.34%
Wisuthsarewong W [92]	2000	Thailand	0~12	-	2361	97	4.11%
Yang XQ [93]	2001	China	15~78	1990~2000	735	6	0.82%
Li GP [94]	2003	China	-	2001~2002	7796	72	0.92%
Ogunbiyi AO [95]	2004	Nigeria	-	1994~1998	1091	51	4.67%
Onayemi O [96]	2005	Nigeria	all	1999~2001	2611	25	0.96%
Nnoruka EN [97]	2005	Nigeria	0~73	1999~2001	2871	91	3.17%
Yang QY [98]	2007	China	60~93	2005~2006	599	2	0.33%
El-Essawi D [62]	2007	USA	20~80	-	207	5	2.42%
Tamer E [99]	2008	Turkey	0~16	2004~2006	6300	91	1.44%
Taylor A [100]	2008	USA	≥12	-	140	1	0.71%
Ayanlowo O [101]	2009	Nigeria	-	2003~2006	6645	186	2.80%
Poojary SA [102]	2011	India	-	2002~2008	33252	204	0.61%
Furue M [103]	2011	Japan	all	2007~2008	67448	1134	1.68%
Muteba Baseke C [104]	2011	Congo	all	2000~2010	14195	204	1.44%
Zhang LJ [105]	2012	China	8~76	2009~2011	1439	13	0.90%
Al-Refu K [106]	2012	Jordan	0~12	2-year period	2000	71	3.55%
Kumar S [107]	2014	India	all	2012	443	44	9.98%
Su WL [108]	2014	China	≥60	2011~2013	1094	2	0.18%

### The results of pooled meta-analysis

**Fig 2:**
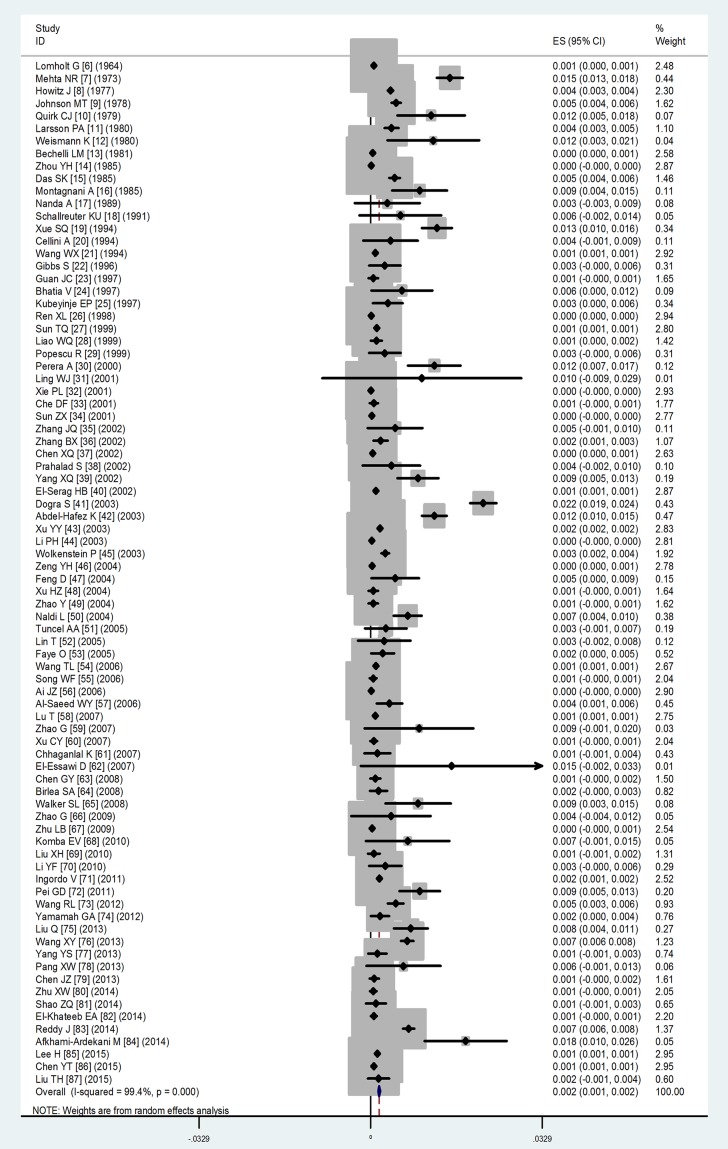
Forest plot of prevalence from population- or community-based studies. Forest plot of prevalence of vitiligo from population- or community-based studies from 1964 to 2015.

**Fig 3:**
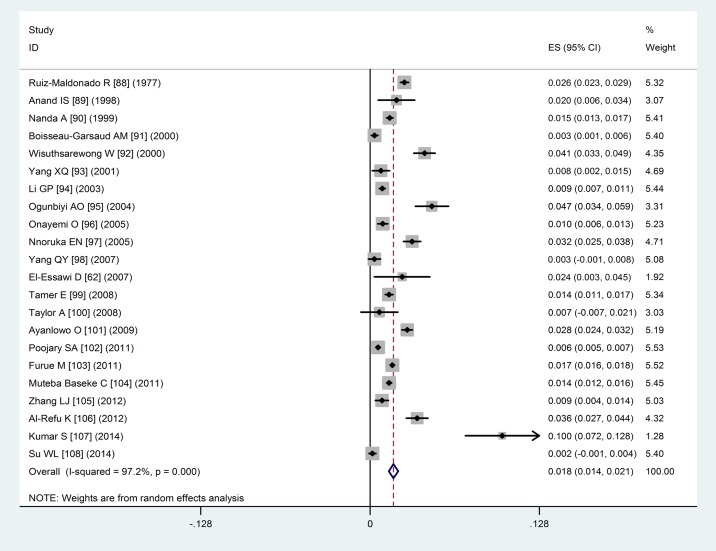
Forest plot of prevalence from hospital-based studies. Forest plot of prevalence of vitiligo from hospital-based studies from 1977 to 2014.

Based on the results of random-effects method, the prevalence of vitiligo from population- or community-based studies was 0.2% (95%CI: 0.1%–0.2%) and from hospital-based studies was 1.8% (95%CI: 1.4%–2.1%). The forest plots of vitiligo prevalence were shown in Figs 2 and 3.

### The subgroup analyses of population- or community-based studies (Table 2)

**Table 2:** Prevalence of vitiligo stratified by different factors.

	Stratified factors	No. of Studies	Prevalence rate	Lower limit	Upper limit	Heterogeneity I^2^ (%)	*P* from test of heterogeneity	Model
Population or community-based studies								
Area								
	Asia	57	0.001	0.001	0.002	99.50%	0	Random
	Africa	7	0.004	0.001	0.007	93.10%	0	Random
	America	5	0.002	0.001	0.004	94.80%	0	Random
	Europe	11	0.004	0.002	0.005	83.90%	0	Random
	Oceania	1	0.012	0.005	0.018	-	-	Random
	Atlantic	1	0.001	0	0.001	-	-	Random
Years								
	1964~1980	7	0.006	0.004	0.009	97.30%	0	Random
	1981~1985	4	0.002	0.001	0.003	96.20%	0	Random
	1986~1990	1	0.003	-0.003	0.009	-	-	Random
	1991~1995	4	0.006	-0.001	0.013	95.40%	0	Random
	1996~2000	9	0.001	0.001	0.002	93.00%	0	Random
	2001~2005	23	0.002	0.002	0.003	97.10%	0	Random
	2006~2010	17	0.001	0	0.001	77.90%	0	Random
	2011~2015	17	0.002	0.002	0.002	99.80%	0	Random
Gender								
	male	30	0.002	0.002	0.003	94.10%	0	Random
	female	18	0.005	0.004	0.006	95.00%	0	Random
Age								
	0~19	26	0.002	0.002	0.003	95.80%	0	Random
	20~39	8	0.002	0.001	0.003	83.30%	0	Random
	40~59	7	0.004	0.003	0.006	90.20%	0	Random
	≥60	7	0.007	0.003	0.01	95.30%	0	Random
Hospital-based studies								
Area								
	Asia	13	0.016	0.011	0.02	97.50%	0	Random
	Africa	5	0.025	0.016	0.034	95.70%	0	Random
	America	4	0.015	-0.001	0.031	97.60%	0	Random
Years								
	1964~1980	1	0.026	0.023	0.029	-	-	Random
	1996~2000	4	0.019	0.007	0.031	97.10%	0	Random
	2001~2005	5	0.02	0.01	0.029	94.70%	0	Random
	2006~2010	5	0.015	0.005	0.025	94.10%	0	Random
	2011~2015	7	0.016	0.01	0.022	98.50%	0	Random
Gender								
	male	4	0.011	0.005	0.017	94.20%	0	Random
	female	4	0.013	0.007	0.02	94.60%	0	Random
Age								
	0~19	7	0.024	0.018	0.029	93.00%	0	Random
	20~39	1	0.014	0.012	0.016	-	-	Random
	40~59	1	0.015	0.013	0.017	-	-	Random
	≥60	3	0.008	-0.005	0.02	98.50%	0	Random

The vitiligo prevalence of different areas were 0.1% (0.1%, 0.2%) in Asia, 0.4% (0.1%, 0.7%) in Africa, 0.2% (0.1%, 0.4%) in America, 0.4% (0.2%, 0.5%) in Europe, 1.2% (0.5%, 1.8%) in Oceania (only one study) and 0.1% (0%, 0.1%) in Atlantic, respectively.

When stratified by publication years, the prevalence of vitiligo was 0.6% (0.4%, 0.9%) before the 80s. It decreased to 0.2%~0.3% in the 80s. The prevalence rebounded to 0.6% (-0.1%, 1.3%) in the first half of 90s. After that, the prevalence drastically decreased and maintained at a low level of 0.1%~0.2%.

The subgroup analysis stratified by gender showed that vitiligo attacked 0.2% (0.2%, 0.3%) males in contrast to 0.5% (0.4%, 0.6%) females.

Pooled prevalence of age-groups in 0~19 years, 20~39 years, 40~59 years and ≥60 years were 0.2% (0.2%, 0.3%), 0.2% (0.1%, 0.3%), 0.4% (0.3%, 0.6%) and 0.7% (0.3%, 1.0%), respectively. The prevalence in the ≥60 years age-group was the highest of the four age categories, and the prevalence of vitiligo increased with age gradually.

### The subgroup analyses of hospital-based studies (Table 2)

With regard to hospital-based studies, the prevalence of vitiligo was as high as 2.5% (1.6%, 3.4%) of Africa, compared with 1.6% (1.1%, 2.0%) of Asia and 1.5% (-0.1%, 3.1%) of America.

Before the 80s, the prevalence of vitiligo was 2.6% (2.3%, 2.9%). The data of the 80s and the first half of 90s were not available. The prevalence of the latter half of 90s and the first 5 years of 21^st^ century were 1.9% (0.7%, 3.1%) and 2.0% (1.0%, 2.9%), respectively. In recent 10 years, the prevalence has decreased to 1.5%~1.6%. It has showed a decreased trend from 60s till now.

The prevalence of males was 1.1% (0.5%, 1.7%) in contrast to 1.3% (0.7%, 2.0%) of females.

The prevalence of age-groups in 0~19 years, 20~39 years, 40~59 years and ≥60 years were 2.4% (1.8%, 2.9%), 1.4% (1.2%, 1.6%), 1.5% (1.3%, 1.7%) and 0.8% (-0.5%, 2.0%), respectively. The highest prevalence was observed in 0~19 years and the overall prevalence showed a gradually decreased trend with age increment.

### Publication Bias

**Fig 4:**
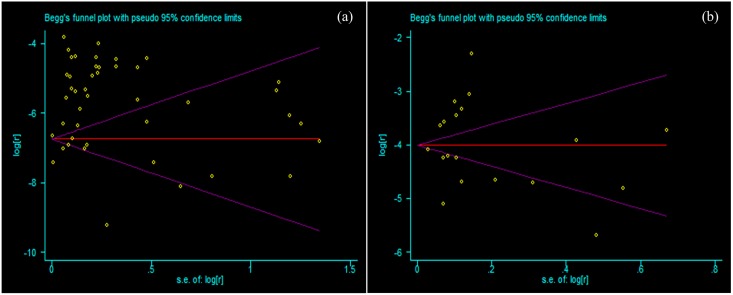
Funnel plot assessing publication bias: (a) population- or community-based published studies; (b) hospital-based published studies. Funnel plot assessing publication bias in the prevalence of vitiligo from: (a) 82 population- or community-based published studies; (b) 22 hospital-based published studies.

There was significant publication bias in population- or community-based studies (t = 2.31, *P* = 0.026), while no significant publication bias existed in hospital-based studies (t = 0.47, *P* = 0.643). The funnel plot of publication bias was shown in Fig 4.

## Discussion

To our knowledge, this is the first meta-analysis examining the prevalence of vitiligo. The results of this study showed that the pooled prevalence of 82 population- or community-based studies was 0.2% and of 22 hospital-based studies was 1.8%. The latter data derived from hospital-based surveys was obviously high.

Although vitiligo occurs worldwide, it is known that the reported prevalence of vitiligo is various. Prevalence distributions might differ in areas. In the included population- or community-based studies, the lowest prevalence was in Asia and Atlantic, the second-highest in Africa and in Europe, and the highest in Oceania. But only 1 study was included, the result of Oceania was not definite. In hospital-based studies, prevalence was the lowest in America and the highest in Africa. So we could draw a common conclusion from the two types of studies that Africa had a high prevalence of vitiligo. These results were in accordance with previous studies that vitiligo frequently occurred in darker-skinned individuals [109]. However, differences did exist in various reports. We suspect the differences that vitiligo is more prevalent in some geographic areas may result from the following factors. Firstly, different skin types and ethnic groups may play important roles in the discrepancy of the prevalence among different areas. Environmental conditions as well as genetic factors may solely or synergistically contribute to the various prevalence distribution in different geographic areas [7]. Secondly, the populations in many surveys were ethnically and culturally diverse such as the survey in USA. Several generations of immigrants or various population's lifestyles in this region might contribute to these differences. Thirdly, unbalanced number of included studies in geographic regions might compromise accurate and sufficient information for heterogeneity. Studies were extremely more conducted in Asia, while only 1 study was conducted in Oceania or Atlantic. Lastly, small amounts of participants in some included studies may contribute to imprecise estimates.

In general, the prevalence of vitiligo showed a relatively decreased trend with increase in the times. Especially, it has remained at a low level in recent two decades in both population- or community-based studies and hospital-based studies. The association between vitiligo and its autoimmune diseases, such as autoimmune thyroid diseases, psoriasis, pernicious anemia, Addison’s disease et al has been frequently described in the literatures. As vitiligo may accompany with other diseases or disorders, we assume that the decreasing prevalence may be beneficial from development of diagnostic tools or improvement of screening programs or therapeutic methods of vitiligo-related diseases or disorders. The prevalence went up to 0.6% in the first half of 90s in population- or community-based studies. We found there was a literature written by Xue SQ about skin diseases of workers and technical personnel in Taigang company. The prevalence of vitiligo of this community population reached 1.27%, higher than other papers in this period. Chemical elements or decolorization may result in the increase of patients with vitiligo. The exact etiology and pathogenesis of vitiligo have not been completely unraveled. It involves a series of known and unknown environmental factors or immunological factors acting over time.

Our results also demonstrated that the pooled prevalence of vitiligo was slightly higher in females than in males, both for the population- or community-based studies (0.5% compared with 0.2%) and the hospital-based studies (1.3% compared with 1.1%). This result was different from previous literatures, which revealed that male and female patients were affected equally by vitiligo [4, 58, 110] or men were more affected than women [111]. Women usually incline to concern about pigmentation changes of their skin and the impact on their social life, and women may be more diligent in seeking treatment. This could be a possible reason for the greater number of female patients in this study [112].

Besides, this study revealed that the prevalence of vitiligo increased with age in population- or community-based studies, increasing gradually from 0.2% in the 0~19 years age-group to 0.7% in the ≥ 60 years age-group. Similar results have been found in some previous studies [8, 58]. The increase possibly correlates to a cumulative effect, because vitiligo is a long-lasting disease and is life-long in most patients. However, in hospital-based studies, the prevalence in the 0~19 years age-group was higher than that in the ≥ 60 years age-group. Youngers may occupy more important position in the family and parents will take them to see a doctor as soon as they find the children’s conditions. In contrast, some elderly patients do not pay greater awareness of their appearance and will not see doctors unless necessary.

Despite we have conducted a comprehensive searching of the epidemiology of vitiligo, several limitations should be considered in this meta-analysis. The available publications/studies were from 31 countries. The data of unavailable countries are required to reflect the wide variation. Some characteristics of the patients, such as clinical types, site or age of onset, risk factors, etc., were not included in the subgroup analyses. These might exert an important influence on the prevalence of vitiligo. Another possible limitation of this study was related to publication bias. The result from Egger’s test showed an evidence of publication bias in population- or community-based studies. It may result from unbalanced number of studies and year of publications. For example, the number of included studies of 2001~2005 was 23, in contrast, the number of 1986~1990 was only 1. Finally, some included studies had noted methodological flaws, especially related to selection and recruitment of samples. Special subjects, such as soldiers, teachers, miners and other professional workers, participating in the investigations could not be representative of other samples. Control group with other diseases such as diabetes was also selected in some studies. As a result, the estimates of prevalence may have been influenced in unpredictable ways and need continuous perfectibility for verifying our conclusion.

In conclusion, we investigated a worldwide prevalence of vitiligo with population- or community-based and hospital-based data. A relatively high prevalence of vitiligo was found in Africa area and in female patients. The prevalence has maintained at a low level in recent years. It showed an inverse trend with age increment in the two types of studies. The current study provided a basic result for further studies. Future researches should be done to find key factors that contribute to the prevalence.

## Supporting Information

S1 File Figure legends. (DOCX)

S1 Table PRISMA 2009 checklist. (DOC)
