# Supplementary material for: The Prevalence of Vitiligo: A Meta-Analysis
Source: PLoS One. 2016 Sep 27;11(9):e0163806. doi: 10.1371/journal.pone.0163806 (PMC5038943; doi:10.1371/journal.pone.0163806)
Supplement: S1 File — (DOCX) [file pone.0163806.s001.docx]

**Figure legends**

Figure 1. Flow diagram of the study selection process.

Figure 2. Forest plot of prevalence of vitiligo from population- or community-based studies from 1964 to 2015.

Figure 3. Forest plot of prevalence of vitiligo from hospital-based studies from 1977 to 2014.

Figure 4. Funnel plot assessing publication bias in the prevalence of vitiligo

(a) 82 population- or community-based published studies; (b) 22 hospital-based published studies.
